# Upregulated expression of serum exosomal hsa_circ_0026611 is associated with lymph node metastasis and poor prognosis of esophageal squamous cell carcinoma

**DOI:** 10.7150/jca.50548

**Published:** 2021-01-01

**Authors:** Shuang Liu, Zheng Lin, Wenqing Rao, Jing Zheng, Qianwen Xie, Yulan Lin, Xi Lin, Huilin Chen, Yuanmei Chen, Zhijian Hu

**Affiliations:** 1 Department of Epidemiology and Health Statistics, Fujian Provincial Key Laboratory of Environment Factors and Cancer, School of Public Health, Fujian Medical University, Fuzhou 350122, China.; 2 Department of Statistics Office, Zhangzhou Affiliated Hospital of Fujian Medical University, Zhangzhou 363000, China.; 3 Department of Radiation Oncology, Anxi County Hospital, Quanzhou 352400, China.; 4 Department of Thoracic Surgery, Fujian Cancer Hospital & Fujian Medical University Cancer Hospital, Fuzhou 350014, China.; 5 Key Laboratory of Ministry of Education for Gastrointestinal Cancer, Fujian Medical University, Fuzhou 350122, China.

**Keywords:** esophageal squamous cell carcinoma, exosome, hsa_circ_0026611, lymph node metastasis, prognosis

## Abstract

**Background:** To identify the diagnostic and prognostic values of serum exosomal hsa_circ_0026611 in esophageal squamous cell carcinoma (ESCC).

**Methods:** ESCC serum exosome global circRNA expression was detected using a circRNA microarray. The expression levels of candidate serum exosome circRNAs were detected by quantitative polymerase chain reaction (qRT-PCR). A receiver operating characteristic curve (ROC) was generated to confirm the diagnostic value. Survival data and their differences were observed by the Kaplan-Meier method and log-rank tests. The Cox regression analysis was employed to identify prognostic factors.

**Results:** The expression levels of serum exosomal has_circ_0026611 in ESCC with lymph node metastasis were significantly higher than those in ESCC without lymph node metastasis (*P* =0.001). In addition, serum exosomal hsa_circ_0026611 expression could be used as a significant parameter to discriminate between non-lymph node-metastatic and lymph node-metastatic ESCC with an area under curve (AUC) of 0.724 (95% CI: 0.604~0.865). The multivariate Cox regression analysis indicated that survival was poor in patients with high serum exosomal hsa_circ_0026611 expression levels compared to those with low serum exosomal hsa_circ_0026611 levels (for OS, HR [95% CI]: 3.79 [1.27, 11.29], for DFS, HR [95% CI]: 2.77 [1.06, 7.22]).

**Conclusion:** Serum exosomal hsa_circ_0026611 expression is significantly upregulated in ESCC with lymph node metastasis and is a predictor of ESCC prognosis.

## Introduction

Esophageal cancer is the seventh most common cancer worldwide and the fourth most common cause of cancer death, with a mortality rate of 16.64/100,000 in China 1,2. There are two major subtypes of esophageal cancer: esophageal squamous cell carcinoma (ESCC) and esophageal adenocarcinoma (EAC). ESCC is the most common type of esophageal cancer, accounting for more than 90% of patients in China 3. It is generally considered that lymph node metastasis (LNM) is an independent prognostic factor for ESCC. The 5-year survival rate for ESCC with early lymph node metastasis is less than 10%, while the proportion of patients with early-stage ESCC is higher than 90% 4. Therefore, treatments to inhibit ESCC metastasis and recurrence are prioritized. However, the mechanisms involved in the development of the lymph node metastasis of ESCC are still unknown. Therefore, novel biomarkers for lymph node metastasis and prognosis of primary ESCC are urgently needed.

Exosomes are extracellular vesicles, and these nanoparticles have a size of 50-140 nm and carry specific cargos, including DNA, proteins, lipids and RNA 5,6. Cancer-derived exosomes may mediate the propagation of cancer by transferring pathogenic factors from cancer cells to healthy cells. Therefore, release of exosome contents into recipient cells can modulate the fate of the recipient cells 7. Studies have shown that tumor-derived exosomes may contribute to tumor metastasis 8. For instance, Lucia *et al.* 9 demonstrated that exosomal miRNAs stimulated the secretion of the proinflammatory cytokine IL-6 from tumor-associated macrophages in a TLR7/8-dependent manner, which in turn promoted liposarcoma cell invasion and metastasis. These studies have provided evidence of the importance of tumor-derived exosomes in the metastasis of tumor cells and provide new directions for the treatment of metastases.

Circular RNAs (circRNAs) represent a large class of non-coding RNAs that are widespread in the cytoplasm of eukaryotic cells and differ from long noncoding RNAs and microRNAs in that they do not have 5′ and 3′ end structures and exist as covalently closed cyclic structures 10. CircRNAs have higher stability and conservation than linear RNAs 11. CircRNAs can exist in exosomes and blood plasma because of their stability. Studies have confirmed that circRNAs are enriched by at least 2-fold in exosomes compared to exosome-producing cells 12. A growing number of studies have revealed that circRNAs play a critical role in the development of cancer 13-15, including ESCC 16. However, to date, there has been no study on the association between exosomal circRNA expression and lymph node metastasis in ESCC.

In this study, we isolated exosomes from serum ESCC samples and normal control individuals. RNA samples were further profiled to determine whether exosome circRNAs could serve as a novel and specific potential biomarker for ESCC.

## Materials & Methods

### Patients & samples

ESCC serum samples were obtained from newly diagnosed ESCC patients who underwent surgery between December 2014 and August 2017 in Fujian Cancer Hospital & Fujian Medical University Cancer Hospital. All enrolled patients underwent face-to-face interviews by trained interviewers using a standardized questionnaire. We recruited subjects who underwent surgery at Fujian Cancer Hospital & Fujian Medical University Cancer Hospital. Pathological results were available for all patients with ESCC, and the results were confirmed by pathologists in this hospital. We excluded patients with other cancers in this study. All patients did not receive chemotherapy or radiotherapy before they underwent surgery. Tumor stages were identified according to the American Joint Committee on Cancer tumor node metastasis (TNM) staging criteria. In preliminary screening, the exosomal circRNA was extracted from ESCC patients with lymph node metastasis (n=4), sex- and age-matched (±5 years) ESCC patients without lymph node metastasis (n=4) and normal control individuals (n=4). In this study, the expression profiles of exosomal circRNA were analyzed using microarray analysis and compared in terms of three groups (A group: ESCC with lymph node metastasis vs ESCC without lymph node metastasis; B group: ESCC with lymph node metastasis vs normal control individuals; C group: ESCC with lymph node metastasis vs postoperative ESCC with lymph node metastasis).

Samples were categorized into the ESCC with lymph node metastasis group (LNM group) if they came from patients who had lymph node dissection ≥ 10 and lymph node metastasis ≥ 1. Samples were categorized into the ESCC without lymph node metastasis group (non-LNM group) if they came from patients who had lymph node dissection ≥ 10 and lymph node metastasis =0. Blood sampling was performed before surgery. In addition, postoperative blood samples were also collected from patients with ESCC. Samples from normal control individuals were retrieved from four physical examinations, which excluded cancer and inflammation, in the same hospital. A total of 69 serum samples from patients with ESCC were collected. The clinical characteristics of the ESCC patients are summarized in Supplement Table 1. Among the patients, there were 35 patients with lymph node metastasis and 34 patients without lymph node metastasis. This study was approved by the Ethical Committee of Fujian Medical University (number: 201495), and the subjects provided informed consent.

### Blood processing, serum exosome isolation and confirm exosome

Blood was collected in a serum clot activator tube and before centrifugation at 2000 r/min for 10 mins. And 2-ml of serum was stored at -80 °C for later use. Serum derived exosomes were isolated from 600 μL of serum using the Exoquick (SBI, America) as per the manufacturer's instructions. Briefly, serum samples were spun at 3000× g for 15 minutes to remove cells and debris. Next, 200 μL of exosome isolation reagent was added to clarified supernatants and samples were incubated at 4°C for 30 min. The precipitated exosomes were recovered by centrifugation at 1500× g for 30 minutes at room temperature. Exosome pellets were resuspended in 60 μL of PBS. The exosomes size was confirmed by using a transmission electron microscopy (HITACHI, Japan).

### Western blot

To further validate our exosome preparations, western blot analyses were conducted for exosomal markers CD63 and CD81. Samples of exosomes were washed and resuspended in RIPA lysis buffer (Bi Yuntian, Hefei, China) with the protease inhibitor mixture. The exosome of proteins was analyzed by Western blot as described. The PVDF membranes with transferred proteins were incubated with primary antibodies at 4ºC overnight and HRP-conjugated secondary antibodies (SBI, America) at room temperature for 1 h. Chemiluminescence reagents (Wabcan, Shanghai, China) was used to visualize the bands. The membrane was observed completing the developing in a darkroom and photography.

### Exosome circRNAs Extraction

CircRNAs were isolated from exosomes using the Trizol Reagent and Protein Isolation Kit (Life Technologies, America) according to the manufacturer's protocol. The purity and concentration of all RNA samples were quantified spectrophotometrically using the NanoDrop ND-1000 system (NanoDrop, Thermo, America) the total exosome RNA of all samples over 30 ng.

### CircRNA Microarray Analysis

The sample preparation and microarray hybridization were performed based on Agilent circRNA (Agilent Technologies Inc.). Briefly, total RNA from each sample was amplified and transcribed into fuorescent cRNA utilizing random primer according to Agilent circRNA protocol. The labeled cRNAs were hybridized onto the Agilent circRNA Array. After having washed the slides, the arrays were scanned by the Agilent scanner (G2565CA). Scanned images were then imported into Agilent Feature Extraction (v10.7) for grid alignment and data extraction. EdgeR package (R 3.4.4) was used to normalize the data and perform analysis to determine differential expression among the circRNAs.

### Quantitative real-time PCR

The expression of circRNAs was measured using quantitative polymerase chain reaction (qRT-PCR) SybrGreen (Takara, Tokyo, Japan) in a Real time PCR System (Thermo, America). Sequences of serum exosomal hsa_circ_0026611 primers sequences as follows, 5′- CACTCCCCACATTCCCACCT -3′; reverse, 5′- AGATTCGTATGCGGACGGGT -3' (the primers sequences of hsa_circ_0126925, hsa_circ_0133794 and hsa_circ_0081144 were listed in Supplement Table 2). The RNA levels were normalized to glyceraldehyde-3-phosphate dehydrogenase (GAPDH). The primer sequences of GAPDH were the following, 5- GAACGGGAAGCTCACTG -3′and 5′- GCCTGCTTCACCACCTTCT -3′. These primers were synthesized by Invitrogen (Guangdong, China). The reaction conditions were as follows: 37 °C for 15 min, and 50 cycles of 95 °C for 30 s and 55 °C for 34 s. The expression levels were calculated by the 2^-ΔCt^ method.

### Gene ontology and pathway analysis

To detect the underlying biological pathways in ESCC, we conducted GO and KEGG pathway analyses based on all differentially expressed circRNAs using KOBAS 3.0(KOBAS 3.0, http://kobas.cbi.pku.edu.cn/), and significant correlations between target genes and their associated functions and pathways were assessed based on a threshold of *P* < 0.05.

### Follow-up

The overall survival (OS) was measured from the date of diagnosis to the date of death due to any cause or the date of the most recent follow-up. Disease-free survival (DFS) was measured from the date of diagnosis to the date of disease relapse, death due to any cause, or the most recent follow-up. All patients were followed every 3 months for the first year, every 6 months for the next two years, and then annually. The median follow-up time was 17.3 months (range from 1 to 43 months) in this study. The end of the follow-up was 31^st^ November 2018.

### Statistical analysis

Analysis of the differentially expressed circRNAs was performed using the EdgeR package in R (R 3.4.4). The differentially expressed circRNAs were identified through fold change filtering. Standard selection criteria for identifying the differentially expressed circRNAs were established as fold change (FC) >2 or <0.5 and *P* <0.05. A hierarchical clustering analysis was performed using R software (R 3.4.4). The Wilcoxon test was applied to evaluate clinical characteristics and their association with circRNAs expression. ROC curves and the area under the ROC curve were used to evaluate the value of the identified serum exosomal hsa_circ_0026611 in detecting ESCC with lymph node metastasis. The survival rate was calculated using the Kaplan-Meier method, and the comparison of two groups was performed using the log-rank test. We used the Cox regression analysis to investigate prognostic factors for ESCC. R software (R 3.4.4) was used for statistical analyses and to construct graphs. A two-sided *P* value < 0.05 was considered statistically significant.

## Results

### Characterization of isolated serum exosomes

As nanoparticles, exosome microvesicle clusters have a size of 20-80 nm in the serum and exhibit round or elliptical vesicular membranes (Supplement Figure 1). To validate our exosome preparations, we analyzed five ESCC serum samples by western blot for the exosomal markers CD63 and CD81. The expression of CD63 and CD81 was specifically observed as a dual band in isolated exosomes (Supplement Figure 1). These findings showed that exosome enrichment was observed in patient serum that had been treated with the ExoQuick isolation reagent.

### Screening of differentially expressed serum exosome circRNAs

**Figure 1:**
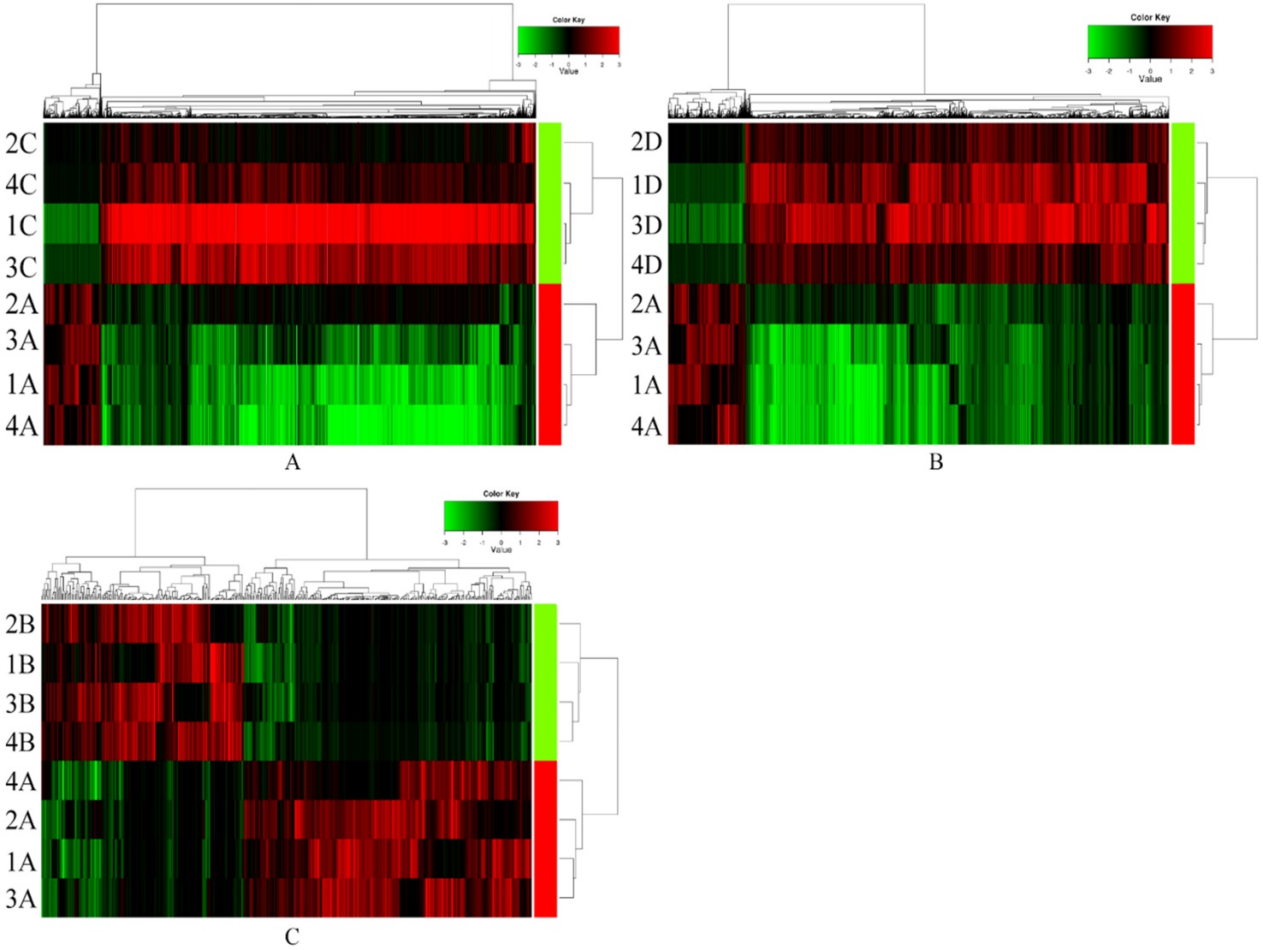
CircRNAs expression signature of ESCC with non-lymph node metastasis, normal control individuals and postoperative ESCC with lymph node metastasis compared to ESCC with lymph node metastasis samples. Each row represents a sample (red color for ESCC with lymph node metastasis (1-4 A), green color for postoperative ESCC with lymph node metastasis (1-4 B), ESCC with non-lymph node metastasis (1-4 C) and normal control individuals (1-4 D)) and each column represents the expression of a circRNA. “Red” indicates high relative expression, and “green” indicates low relative expression of circRNAs.

In preliminary screening, serum exosomal circRNAs (exo-circRNAs) were extracted from samples of ESCC with lymph node metastasis (n=4), samples from sex- and age-matched (± 5 years) ESCC patients without lymph node metastasis (n=4) and samples from normal control individuals (n=4). The comparison of serum exosomal circRNA expression between samples of ESCC with lymph node metastasis and samples of ESCC without lymph node metastasis showed 1101 significantly upregulated circRNAs and 8384 downregulated circRNAs (Figure 1A). Overall, 18,000 exosomal circRNAs were differentially expressed between samples from patients with ESCC with lymph node metastasis and those from normal control individuals, including 2293 upregulated and 15,707 downregulated circRNAs (Figure 1B). A total of 439 differentially expressed exosomal circRNAs (258 significantly upregulated circRNAs and 181 significantly downregulated circRNAs) were identified between samples from preoperative ESCC with lymph node metastasis and paired postoperative ESCC samples (Figure 1C). The distributions of the circRNA profiles were visualized using heatmaps (Figure 1).

Next, we identified those exosomal circRNAs that warranted further study: 1) Those circRNAs that showed overlapping differential expression in all three comparison groups (106 circRNAs) were selected using a Venn diagram, including 50 significantly upregulated circRNAs and 56 downregulated circRNAs. 2) In addition, with criteria of fold change ≥3.0 and *P* <0.05, we selected only upregulated circRNAs for further study. 3) For qRT-PCR analysis of circRNAs, it is necessary to design primers on both sides of the back-splicing site or across the site and to design primers with selectable sequences and locations. This is quite different from designing primers for qRT-PCR analysis of conventional RNA. As such, the circRNAs selected for further experiments needed to allow the design of appropriate specific primers, and the length of the gene needed to be suitable for subsequent qRT-PCR experiments. According to these criteria, we identified exosomal hsa_circ_0026611, exosomal hsa_circ_0126925, exosomal hsa_circ_0133794 and exosomal hsa_circ_0081144 for further study.

### Expression of the four serum exosomal circRNAs in ESCC

qRT-PCR was used to detect the expression levels of the four serum exosomal circRNAs in ESCC. In the initial verification process with 20 ESCC samples (including 10 samples from ESCC with lymph node metastasis and 10 samples from ESCC without lymph node metastasis), the serum levels of exosomal hsa_circ_0026611 and exosomal hsa_circ_0081144 were significantly different between the two groups (*P* <0.01 and *P* =0.04, respectively) (Supplement Table 3). In the extended verification process with 69 ESCC samples (including 35 samples from ESCC with lymph node metastasis and 34 samples from ESCC without lymph node metastasis), only the levels of serum exosomal hsa_circ_0026611 were statistically significantly different between the two groups (*P* <0.01) (Supplement Table 4). Thus, we chose serum exosomal hsa_circ_0026611 for further study.

### The association between the expression level of exosomal hsa_circ_0026611 and clinical features

As shown in Table 1, we further compared the serum exosomal hsa_circ_0026611 expression level with various clinical factors. Among the various clinical factors, we found a significant association between serum exosomal hsa_circ_0026611 expression and T stage (*P* =0.032), N stage (*P* =0.001), and postoperative radiotherapy and chemotherapy (*P* =0.037). However, no significant correlations were observed between serum exosomal hsa_circ_0026611 expression and sex, age, tumor location, or histologic grade. These results demonstrated that positive lymph node metastasis was associated with higher exosomal hsa_circ_0026611 expression levels in serum from ESCC patients.

### Serum exosomal hsa_circ_0026611 can predict lymph node metastasis

**Figure 2:**
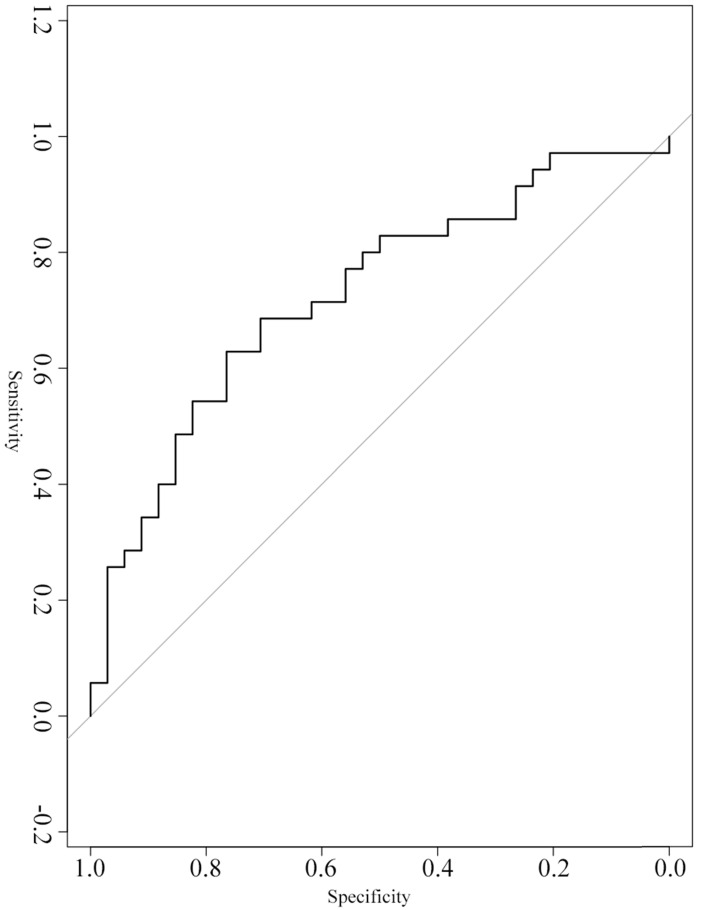
Serum exosomal hsa_circ_0026611 in distinguishing ESCC with lymph node metastasis from ESCC with non-lymph node metastasis.

A ROC curve was constructed to evaluate the diagnostic value of serum exosomal hsa_circ_0026611 in distinguishing ESCC with lymph node metastasis from ESCC without lymph node metastasis (n=69). Our analyses showed that serum exosomal hsa_circ_0026611 expression could be used as a significant parameter to discriminate between non-lymph node-metastatic and lymph node-metastatic ESCC with an AUC of 0.724 (95% CI: 0.604~0.865, Figure 2), and the sensitivity and specificity were 0.800 and 0.529, respectively.

### Potential prognostic values of serum exosomal hsa_circ_0026611

**Figure 3:**
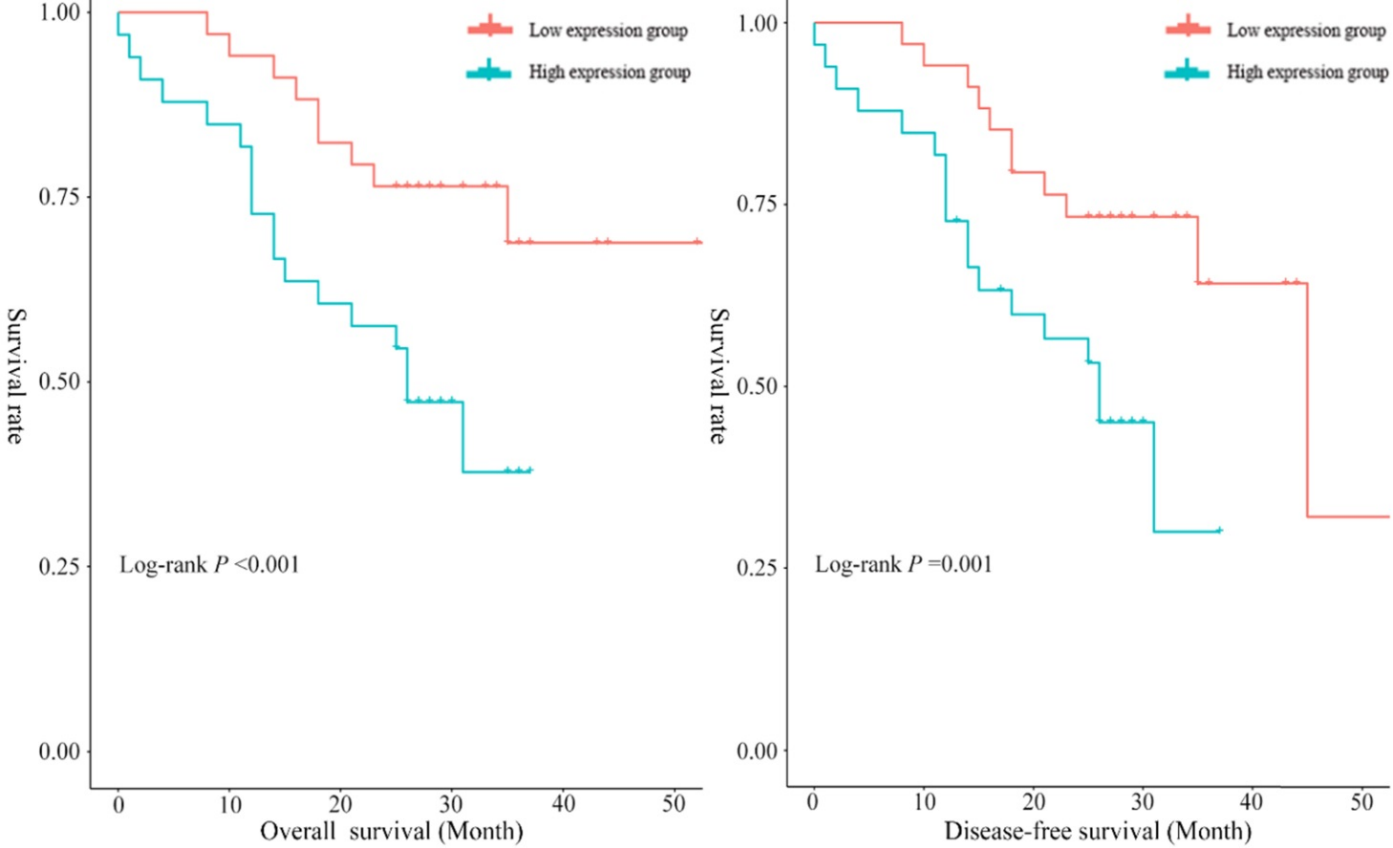
Serum exosomal hsa_circ_0026611 can be an independent prognostic factor to predict OS and DFS.

In the present study, the median overall survival time was 29.12 months (95% CI: 24.68~33.56), and the 1-year and 3-year survival rates were 85.51% (95% CI: 76.50~93.70) and 46.40% (95% CI: 26.30~66.50), respectively. To assess the prognostic significance of serum exosomal hsa_circ_0026611 expression in ESCC, 69 ESCC patients were divided into two groups according to the median expression level of serum exosomal hsa_circ_0026611 (high exosomal hsa_circ_0026611 expression level group vs. low exosomal hsa_circ_0026611 expression level group, n = 35 vs. 34). Kaplan-Meier survival curves were constructed to compare the two groups, and the results are shown in Figure 3. The log-rank test showed that there was a statistically significant difference (for OS, *P* <0.001; for DFS, *P* =0.001.) in the survival between patients with high and low expression levels of serum exosomal hsa_circ_0026611.

To better evaluate the prognostic function of serum exosomal hsa_circ_0026611 expression in ESCC, we used a Cox regression analysis with adjustment for factors including sex, age, tumor location, histologic grade, T stage, postoperative radiotherapy and chemotherapy and serum exosomal hsa_circ_0026611 level. Spearman analysis was used to determine the correlation between serum exosomal hsa_circ_0026611 level and N stage (*P*=0.004). Therefore, N stage was not included in multivariate Cox regression analysis. We found that the patients with high serum exosomal hsa_circ_0026611 expression had a significantly worse survival than those with low expression (for OS, HR [95% CI]: 3.79 [1.27, 11.29], for DFS, HR [95% CI]: 2.77 [1.06, 7.22], Table 2). Collectively, these results suggest that serum exosomal hsa_circ_0026611 may have utility in predicting tumor progression and monitoring for recurrence.

### Functional and pathway enrichment analyses

The top 10 enriched GO terms are shown in Supplement Figure 2. The three most significantly enriched pathways in the GO term analysis were embryonic skeletal system development, organelle lumen, and voltage-gated calcium channel activity. The KEGG pathway enrichment terms are presented in Supplement Figure 2. The results demonstrated that the differentially expressed circRNAs were mainly associated with the other glycan degradation, melanogenesis, and endocytosis super pathways.

## Discussion

Although therapeutic options, including surgery, radiotherapy, and chemotherapy, have advanced in recent years, the prognosis remains unsatisfactory for patients with ESCC 14. Lymph node metastasis is a major contributor to recurrence and prognosis in patients with ESCC. However, the measurement of lymph node size by imaging techniques does not appear to be a reliable indicator of lymph node metastasis due to its limited accuracy 17. Thus, the identification of sensitive biomarkers for the early diagnosis of ESCC lymph node metastasis is urgently needed.

Exosomes transport various biomolecules, including DNA, RNA (including miRNAs and circRNAs), and lipid species. Tumor-derived exosomes can be isolated from serum, and they are proposed to transfer a repertoire of bioactive molecular cargo to recipient cells, which might be involved in tumor invasion 18. Studies have indicated that many circulating miRNAs are passively released from apoptotic and necrotic cells 19, which may not truly reflect the biological changes that occur in these tumor cells. In contrast, exosomes are actively secreted in the peripheral blood by different cell types, including cancer cells 20. Recent studies have found higher expression of miR-451 in the exosomal fraction than the supernatant (fold change = 8.02) in ESCC samples 21. Another study showed that serum exosome miR-1246 expression is specifically upregulated in prostate cancer, but tissue miR-1246 expression is commonly downregulated 22. These findings indicate that molecular cargo (including DNA and RNA) is selectively released into exosomes, leading to high expression of these molecules in serum samples and contributing to their utility as exosomal markers. Numerous studies have investigated whether exosomal miRNAs play functional roles in the development of different cancers. Nevertheless, there are few publications on exosomal circRNAs.

Previous studies demonstrated that circRNAs are abundant and widely expressed in mammals and can display cell type-specific expression 23. Due to their covalently closed cyclic structures, they are not sensitive to ribonucleases and have a longer half-life than linear RNAs 24. Many studies have proven that circRNAs have diverse biological functions and play important roles in cellular activities 25-27. A previous study indicated that circIRAK3 overexpression was present in metastatic breast cancer cells and predictive of breast cancer recurrence 13. Similarly, Luo *et al*. 11 suggested an association between high expression of hsa_circ_0000064 and cell proliferation and migration in lung cancer. In summary, aberrant circRNA expression is associated with prognosis in cancer. Exosomes derived from different tumors contain circRNAs with specific characteristics. Indeed, our analyses suggest a correlation of serum exosomal hsa_circ_0026611 with increasing lymph node metastasis and T stage. Importantly, the median expression of serum exosomal hsa_circ_0026611 was found to be increased in samples of ESCC with lymph node metastasis. These data highlight the potential of this exosomal marker to distinguish ESCC without lymph node metastasis from ESCC with lymph node metastasis. Furthermore, to understand the prognostic significance of serum exosomal hsa_circ_0026611 upregulation in ESCC, multivariate Cox regression analysis was performed to analyze the correlation between serum exosomal hsa_circ_0026611 expression and patient survival. The results illustrated that serum exosomal hsa_circ_0026611 overexpression was closely associated with poor OS and DFS. Hence, we hypothesize that serum exosomal hsa_circ_0026611 might affect prognosis by promoting lymph node metastasis in ESCC. However, the effect of serum exosomal hsa_circ_0026611 on ESCC lymph node metastasis warrants further investigation.

GO terms and KEGG pathway analyses were conducted to analyze the potential functions of differentially expressed circRNAs in ESCC. The KEGG pathway analysis revealed that several significant pathways might be activated in response to ESCC, including the endocytosis pathway. Endocytosis is responsible for bringing ligands, nutrients, plasma membrane proteins, and lipids into the cell interior and removing them from the cell surface 28. Canonical Wnt signaling has been proposed to regulate endocytosis 29. The Wnt/β-catenin signaling pathway plays a critical role in many biological processes, including stem cell maintenance and cell proliferation 30. A recent study demonstrated that BCL11A enhanced tumor formation and cancer cell mobility by activating the Wnt/β-catenin signaling pathway in breast cancer stem cells 31. In addition, in colorectal cancer, Qin *et al*. 32 indicated that knockdown of ZNF281 expression suppressed cell proliferation, migration, and invasion by inhibiting the Wnt/β-catenin pathway. More importantly, the Wnt signaling pathway has been shown to downregulate circRNA expression, which has been reported to have a regulatory role in glioblastoma and prostate cancer 33,34. Therefore, we suspect that circRNAs regulate the Wnt signaling pathway, which modulates endocytosis to affect ESCC. However, further research is warranted to confirm these findings.

There are several limitations to our study. First, only 68 pairs of ESCC serum samples were analyzed in this study. A larger sample size should be used to further confirm our findings. Moreover, it is critical to explore the potential regulatory mechanisms of exosomal hsa_circ_0026611 in the progression of ESCC.

## Conclusions

Serum exosomal hsa_circ_0026611 expression is significantly upregulated in ESCC with lymph node metastasis and might be a predictor of ESCC prognosis.

## Supplementary Material

Supplementary figures and tables.

## Figures and Tables

**Table 1:** Associations between patient's characteristics and serum exosomal hsa_circ_0026611 expression in the ESCC

Variables	n	M (P_25_, P_75_)^*^	*Z*	*P*
**Sex**			1.455	0.146
Female	22	0.62 (0.33,2.94)		
Man	47	1.68 (0.54,2.82)		
Age	69	1.08 (0.47,2.87)	0.016	0.895^#^
**Tumor location**			-0.851	0.395
Upper/middle	40	1.10 (0.53,3.29)		
Distal	29	0.99 (0.41,2.52)		
**Histologic grade**			0.557	0.577
G3/G2	58	0.96 (0.47,2.81)		
G1	11	2.30 (0.35,2.98)		
**T stage**			2.145	0.032
I-II	26	0.60 (0.33,1.89)		
III	42	1.76 (0.50,3.35)		
**N stage**			2.707	0.001
N_0_	34	0.56 (0.34,1.75)		
N_+_	35	2.30 (0.72,3.57)		
**Postoperative radiotherapy and chemotherapy**			2.082	0.037
No	50	0.89 (0.47,2.34)		
Yes	19	2.81 (0.40,4.79)		

*The median (P_25_, P_75_) of serum exosomal hsa_circ_0026611 expression; #Spearman correlations *P* value.

**Table 2:** Univariate and multivariate analysis of overall survival and disease-free survival in ESCC

Variables	Overall survival (OS)		Disease-free survival (DFS)	
	Univariate	Multivariate*	Univariate	Multivariate*
**Sex**				
Female	1.00	1.00	1.00	1.00
Man	2.85 (0.96,8.42)	1.66 (0.50,5.52)	3.64 (1.22,10.28)	2.14 (0.69,6.57)
Age	1.02 (0.96,1.09)	1.00 (0.92,1.06)	1.03 (0.97,1.08)	1.00 (0.94,1.07)
**Tumor location**				
Upper/middle	1.00	1.00	1.00	1.00
Distal	0.94 (0.41,2.16)	0.71 (0.28,1.82)	1.17 (0.55,2.49)	0.94 (0.40,2.21)
**Histologic grade**				
G3/G2	1.00	1.00	1.00	1.00
G1	1.12 (0.38,3.29)	1.10 (0.28,4.26)	1.27 (0.48,3.36)	1.08 (0.33,3.49)
T stage				
I-II	1.00	1.00	1.00	1.00
III	4.83 (1.43,16.37)	5.41 (1.37,21.38)	3.14 (1.18,8.36)	2.77 (0.89,8.63)
**Postoperative radiotherapy and chemotherapy**				
No	1.00	1.00	1.00	1.00
Yes	1.39 (0.57,3.43)	0.63 (0.22,1.78)	1.34 (0.58,3.11)	0.79 (0.30,2.09)
**Serum exosomal has circ_0026611**				
Low group	1.00	1.00	1.00	1.00
High group	4.42 (1.80,10.85)	3.79 (1.27,11.29)	3.76 (1.63,8.70)	2.77 (1.06,7.22)

*Adjusted for sex, age, tumor location, histologic grade, T stage, adjuvant radiotherapy and chemotherapy and serum exosomal hsa_circ_0026611.

## Abbreviations

ESCC: esophageal squamous cell carcinoma
EAC: esophageal adenocarcinoma
qRT-PCR: quantitative real-time PCR
LNM: lymph node metastasis
circRNAs: Circular RNAs
ROC: receiver operating characteristic
AUC: area under curve
OS: overall survival
DFS: disease-free survival
HR: hazard ratio
95% CI: 95% confidence interval
