# Supplementary material for: Upregulated expression of serum exosomal hsa_circ_0026611 is associated with lymph node metastasis and poor prognosis of esophageal squamous cell carcinoma
Source: J Cancer. 2021 Jan 1;12(3):918–26. doi: 10.7150/jca.50548 (PMC7778553; doi:10.7150/jca.50548)
Supplement: Supplementary file 1 — Supplementary figures and tables. [file jcav12p0918s1.pdf]

Supplement table1. The baseline characteristics of ESCC.

**Variables**	**n**	**%/M (P25, P75)**
Sex		
Female	22	31.88
Man	47	68.12
Age	69	63.00(58.00,67.00)
Tumor location		
Upper/middle	40	57.97
Distal	29	42.03
Histologic grade		
G3/G2	58	84.06
G1	11	15.94
T stage		
I-II	26	37.68
III	42	60.87
N stage		
N_0	34	49.28
N_+	35	20.72
Postoperative radiotherapy and chemotherapy		
No	50	72.46
Yes	19	27.54

Supplement table 2. The primers sequences of CircRNAs and GAPDH.

**CircRNAs**	**Forward**	**Reverse**
hsa_circ_0026611	CACTCCCCACATTCCCACCT	AGATTCGTATGCGGACGGGT
hsa_circ_0126925	AGCAAGTCCTAAGCGTGGGC	GCTGCATCCACATCTGCACC
hsa_circ_0133794	AGGTCTTCACCACCATGGCC	TGTGACAAGGTAGCACAGCGA
hsa_circ_0081144	TTCGTGGTCCTCGTGGTGAC	CCAGAAGGACCTCGGCTTCC
GAPDH	GAACGGGAAGCTCACTG	GCCTGCTTCACCACCTTCT

Supplement table 3. The four serum exosomal circRNAs expression levels in ESCC.

**CircRNAs**	**LNM group(n=10)**	**Non-LNM group (n=10)**	**Z**	***P***
	M (P_{25}, P_{75})	M (P_{25}, P_{75})		
hsa_circ_0026611	4.15(2.00,13.54)	0.53(0.44,1.14)	-2.797	<0.01
hsa_circ_0126925	1.74(0.66,3.46)	1.19(0.20,2.70)	-0.756	0.45
hsa_circ_0133794	11.00(0.44,35.74)	0.36(0.03,16.69)	-1.029	0.23
hsa_circ_0081144	7.30(1.37,17.07)	0.46(0.25,4.58)	-2.041	0.04

Supplement table 4. The serum exosomal hsa_circ_0026611、exosomal hsa_circ_0081144 expression levels in ESCC.

CircRNAs	LNM group (n=35)	Non-LNM group (n=34)	Z	*P*
	M (P_{25}, P_{75})	M (P_{25}, P_{75})		
hsa_circ_0026611	2.30(0.72,3.57)	0.56(0.34,1.75)	-3.205	<0.01
hsa_circ_0081144	4.65(0.14,15.33)	1.14(0.25,4.11)	-1.698	0.09

Supplement figures

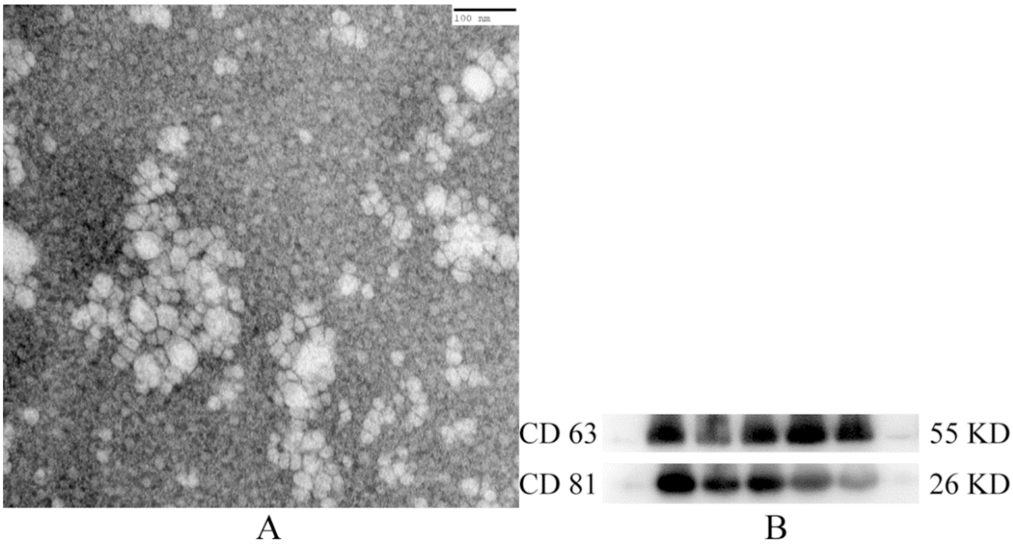

Supplement figure 1. A. Serum exosome were observed under transmission electron microscopy. B. The CD63, CD81 expression levels were analyzed using western blot.

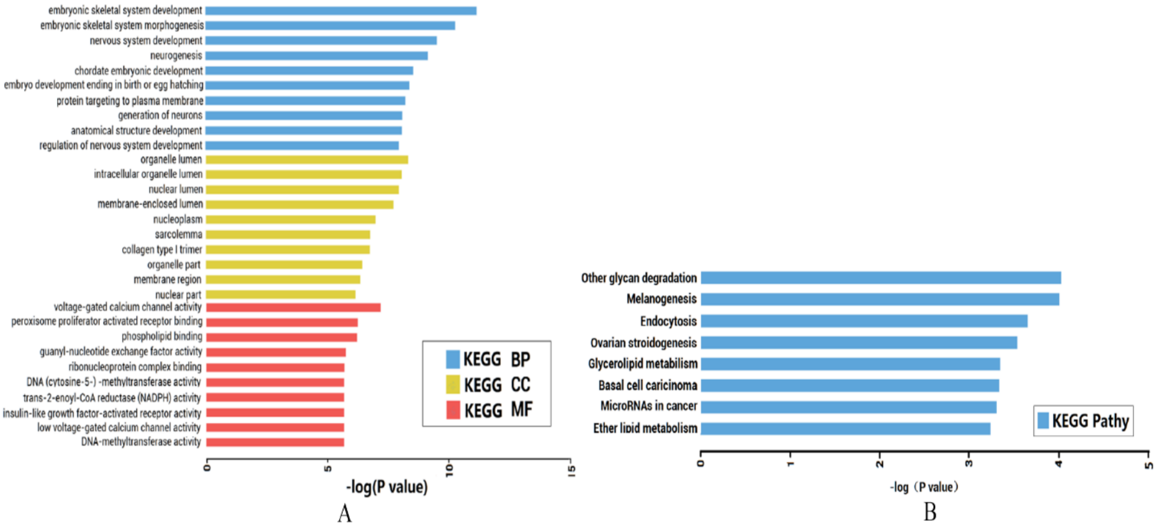

Supplement figure 2. GO functional enrichment analysis and KEGG pathway enrichment analysisof differentially expressed circRNAs.
